# 6-Shogaol reduced chronic inflammatory response in the knees of rats treated with complete Freund's adjuvant

**DOI:** 10.1186/1471-2210-6-12

**Published:** 2006-10-01

**Authors:** Arkene SA Levy, Oswald Simon, Janet Shelly, Michael Gardener

**Affiliations:** 1 Department of Basic Medical Sciences, Pharmacology Section, University of the West Indies, Mona Campus, Jamaica; 2 Department of Basic Medical Sciences, Anatomy Section, University of the West Indies, Mona Campus, Jamaica

## Abstract

**Background:**

6-Shogaol is one of the major compounds in the ginger rhizome that may contribute to its anti-inflammatory properties. Confirmation of this contribution was sought in this study in Sprague- Dawley rats (200–250 g) treated with a single injection (0.5 ml of 1 mg/ml) of a commercial preparation of complete Freund's Adjuvant (CFA) to induce monoarthritis in the right knee over a period of 28 days. During this development of arthritis, each rat received a daily oral dose of either peanut oil (0.2 ml-control) or 6-shogaol (6.2 mg/Kg in 0.2 ml peanut oil).

**Results:**

Within 2 days of CFA injection, the control group produced maximum edematous swelling of the knee that was sustained up to the end of the investigation period. But, in the 6-shogaol treated group, significantly lower magnitudes of unsustained swelling of the knees (from 5.1 ± 0.2 mm to 1.0 ± 0.2 mm, p < 0.002, n = 6) were produced during the investigation period. Unsustained swelling of the knees (from 3.2 ± 0.6 mm to 0.8 ± 1.1 mm, p < 0.00008, n = 6) was also produced after 3 days of treatment with indomethacin (2 mg/Kg/day) as a standard anti-inflammatory drug, but during the first 2 days of drug treatment swelling of the knees was significantly larger (11.6 ± 2.0 mm, p < 0.0002, n = 6) than either the controls or the 6-shogaol treated group of rats. This exaggerated effect in the early stage of indomethacin treatment was inhibited by montelukast, a cysteinyl leukotriene receptor antagonist. Also, 6-shogaol and indomethacin were most effective in reducing swelling of the knees on day 28 when the controls still had maximum swelling. The effect of 6-shogaol compared to the controls was associated with significantly lower concentration of soluble vascular cell adhesion molecule-1 (VCAM-1) in the blood and infiltration of leukocytes, including lymphocytes and monocytes/macrophages, into the synovial cavity of the knee. There was also preservation of the morphological integrity of the cartilage lining the femur compared to damage to this tissue in the peanut oil treated control group of rats.

**Conclusion:**

From these results, it is concluded that 6-shogaol reduced the inflammatory response and protected the femoral cartilage from damage produced in a CFA monoarthritic model of the knee joint of rats.

## Background

6-Shogaol (1-4-hydroxy-3-methoxyphenyl-4-decen-3-one) is one of the major biologically active compounds found in the rhizome of *Zingiber officinale*/ginger [1]. This compound was previously reported to have antipyretic and analgesic effects in addition to inhibitory effect on lipoxygenase activity [2,3]. Its presence in ginger may also contribute to the anti-inflammatory effects associated with the use of powdered ginger [4,5]. To confirm this contribution, 6-shogaol was used in this study as an isolated single compound to determine whether it has anti-inflammatory properties and to demonstrate these properties in an animal model of chronic inflammation.

Many of the animal models used in investigations of chronic inflammation produce polyarthritis after injection of complete Freund's adjuvant (CFA) or other pro-inflammatory agents at the base of the rat tail [6] or in a single foot pad of the rat [7] or a single knee joint of the rat [8]. The polyarthritis developed in these animal models was reported to be dose-dependent such that large doses of the pro-inflammatory agent were more likely to produce inflammation at multiple sites [9] including the corresponding contralateral sites to the injection sites [8]. This type of symmetrical development of polyarthritis in the animal models is similar to human rheumatoid arthritis, osteoarthritis and psoriasis [8], but the mechanisms underlying the inflammatory response at the contralateral sites are still unclear. There is however, evidence of neurological involvement in the development of inflammation at the contralateral site as reported in a review by Shenker et al [8]. Substance P (SP) and calcitonin gene-related peptide (CGRP) were identified as neuropeptides that may be involved in development of contralateral inflammation [8]. These are pro-inflammatory neuropeptides that increase mobilization of leukocytes from the blood and loss of fluid from the circulation (8). The mechanisms of these effects at the contralateral sites may be different from the mechanism induced by the pro-inflammatory agent at the primary injection site. Consequently, experimental data obtained in the polyarthritic model would be complex and difficult to interpret.

To avoid this complexity and to minimise the influence of contralateral inflammation, a monoarthritic model was used to investigate the anti-inflammatory potential of 6-shogaol. The inflammation produced in this monoarthritic model is localized at the site of induction and according to Butler et al [10], it is stable for chronic studies. Our model was developed with the use of a low potency commercial preparation of CFA (compared to preparations made from basic ingredients by other investigators [6,11]) to induce chronic inflammation in the knees of Sprague Dawley rats over a period of 28 days. The mycobacterium content of CFA was reported in several studies to be the inducing agent of chronic inflammation through stimulation of the immune response [12-14]. The resulting pathological features include swelling of the joint due to edema, leukocyte migration into the joint and various degrees of cartilage degradation [6,11,15]. Sfikakis and Tsokos [16] reported that leukocyte migration was facilitated by adhesion molecules in the blood. An example of this type of molecule was identified as soluble vascular cell adhesion molecule-1 (soluble VCAM-1), and it was reported to be produced in increased concentration in the blood of patients with chronic inflammatory diseases such as rheumatoid arthritis [17-19].

These features of chronic inflammation were reproduced in the CFA monoarthritic model used in this study and they provided the basis for evaluating the anti-inflammatory potential of 6-shogaol.

## Results

### Effect of 6-shogaol on edema production in CFA treated rats

**Figure 1:**
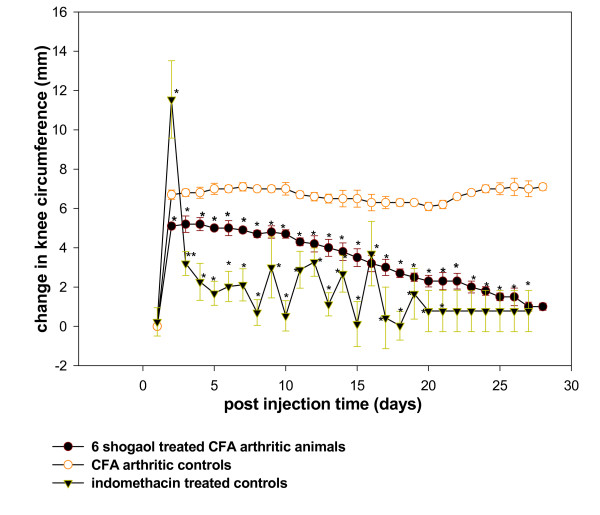
Graph showing the effects of daily oral administration of 6-shogaol (6.2 mg/Kg/day), indomethacin (2 mg/Kg/day) and peanut oil (0.2 ml/day-control) on the development (over 28 days) of edematous swelling of the knee induced by a single dose of CFA (0.5 ml of 1 mg/ml) in the synovial cavity of rats. Each data point represents the mean ± SEM value from 6 animals. Asterisks indicate values significantly different from the peanut oil control (* p ≤ 0.0002, ** p < 0.00008)

In a control group of rats that received peanut oil (0.2 ml, orally) daily for 28 days, a single injection of CFA (0.5 ml of 1 mg/ml) in the synovial cavity of the right knee of each rat produced swelling of the knee due to edema. Maximum swelling of the knee was reached within 2 days of CFA injection and it was measured as a mean increase in knee circumference of 6.7 ± 0.2 mm (fig. 1). This magnitude of swelling was maintained up to the end of the 28 day investigation period (fig. 1). A comparatively lower magnitude of unsustained swelling was produced within 2 days of CFA injection in the 6-shogaol (6.2 mg/kg/day) treated rats. Maximum swelling in this group of rats, obtained after 2 doses of 6-shogaol, was measured as a mean increase in knee circumference of 5.1 ± 0.2 mm which was significantly lower (p < 0.0002, n = 6) than in the control group, and it was progressively reduced after day 2 to reach a minimum of 1.0 ± 0.2 mm on day 28 (fig. 1). Qualitatively similar progressive reduction in mean increase in knee circumference from 3.2 ± 0.6 mm to 0.8 ± 1.1 mm (p < 0.0008, n = 6) was produced after three doses of the standard anti-inflammatory drug indomethacin (2 mg/kg/day). But at the beginning of the treatment, indomethacin did not inhibit the swelling after 2 doses, consequently the maximum swelling, measured as a mean increase in knee circumference of 11.6 ± 2.0 mm was significantly larger (p < 0.0002, n = 6) than in the control and 6-shogaol treated group of rats (fig. 1). This exacerbation of the edematous swelling by indomethacin on day 2 was reduced from 11.6 ± 2.0 mm to 5.0 ± 1.0 mm, when equimolar montelukast (3.4 mg/Kg/day, orally), a cysteinyl leukotriene receptor antagonist, was given in combination with indomethacin from day zero to day three of the treatment period.

### Effect of 6-shogaol on leukocyte infiltration into the synovial cavity of CFA treated rats

**Figure 2:**
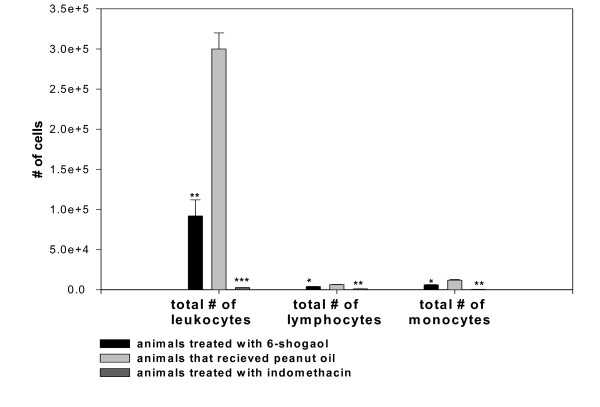
Graph showing the effects of daily administration of 6-shogaol (6.2 mg/Kg/day), indomethacin (2 mg/Kg/day) and peanut oil (0.2 ml/day-control) on total leukocyte numbers and differential counts of monocytes/macrophages and lymphocytes in synovial fluid aspirated from rats with knee inflammation induced by a single dose of CFA (0.5 ml of 1 mg/ml) in the synovial cavity. Each bar represents the mean ± SEM value from 6 animals. Asterisks indicate values significantly different from the peanut oil control (* p < 0.002, ** p < 0.001, *** p < 0.000001).

Total leukocyte concentration in the fluid of the synovial cavity was determined on day 28 when 6-shogaol and indomethacin was most effective in reducing swelling of the knee due to edema production. Figure 2 shows that total leukocyte count on this day in the 6-shogaol and indomethacin treated groups of rats was significantly lower (p < 0.001, p < 0.000001 (respectively), n = 6) than the count in the peanut oil treated control group of rats in which maximum swelling of the knee was still present. Differential counts of the infiltrated leukocytes show (fig. 2) that the 6-shogaol treated group of rats also had significantly lower counts compared to the peanut oil controls for lymphocytes (4.0 ± 0.2 × 10^3 ^versus 6.4 ± 0.3 × 10^3^, p < 0.002, n = 6) and monocytes/macrophages (6.2 ± 0.2 × 10^3 ^versus 12.0 ± 0.7 × 10^3^, p < 0.002, n = 6). Indomethacin treatment also resulted in significantly lower counts (fig. 2) for lymphocytes (1.1 ± 0.03 × 10^3 ^versus 6.4 ± 0.3 × 10^3^, p < 0.001, n = 6) and monocytes/macrophages (0.4 ± 0.01 × 10^3 ^versus 12.0 ± 0.7 × 10^3^, p < 0.001, n = 6).

### Effect of 6-shogaol on VCAM-1 concentration in the blood of CFA treated rats

**Figure 3:**
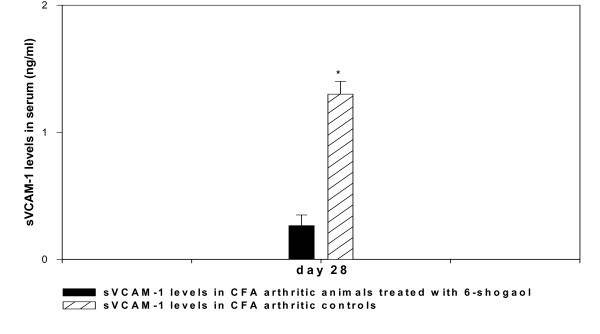
Bar graph showing the concentration of soluble VCAM-1 in the blood of rats on day 28 after treatment with a single dose of CFA (0.5 ml of 1 mg/ml) in the knee, and daily oral doses (for 28 days) of 6-shogaol (6.2 mg/Kg/day) and peanut oil (0.2 ml/day-control). Each bar shows the mean ± SEM value from 6 animals. Asterisk indicates value significantly different from the peanut oil control (* p < 0.001).

Soluble VCAM-1 concentration in the blood was determined on day 28 when 6-shogaol was most effective in reducing swelling of the knee due to edema production. In the peanut oil treated control group, 1.3 ± 0.1 ng/ml of soluble VCAM-1 was detected in the blood compared to 0.3 ± 0.1 ng/ml detected in the blood of the 6-shogaol treated group of rats (fig. 3). These concentrations of soluble VCAM-1 are significantly different (p < 0.001, n = 6), and it is interesting to note that the lower concentration of soluble VCAM-1 in the 6-shogaol treated group of rats was associated with significantly lower infiltration of leukocytes (92 ± 2 × 10^3^, p < 0.001, n = 6) into the synovial cavity compared to the controls (300 ± 2 × 10^3^). Also, the relationship between these two variables showed a strong positive correlation (r = 0.98, p < 0.0005, df = 10) in which the magnitude of leukocyte infiltration was dependent on the concentration of soluble VCAM-1 produced in the blood.

### Effect of 6-shogaol on femoral cartilage morphology of CFA treated rats

**Figure 4:**
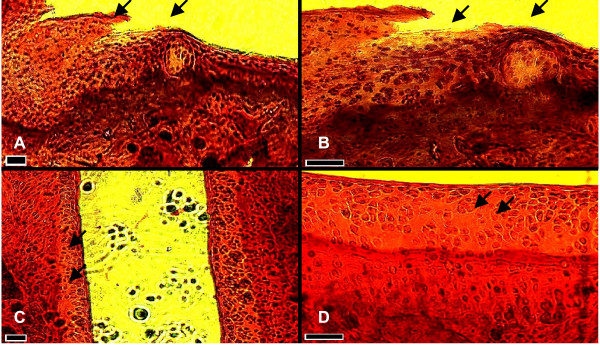
Representative micrographs of histological sections (5 μm) of the knee joint: Panels A and B show damage of the articular cartilage lining the femur of a rat that was given a single dose of CFA (0.5 ml of 1 mg/ml) in the synovial cavity and daily oral dose (0.2 ml) of peanut oil for 28 days. A (Mag × 10), B (Mag × 20), scale bars = 20 μm, Arrows indicate areas of cartilage degradation. Panels C and D show the articular cartilage lining the femur of a rat that was given a single dose of CFA (0.5 ml of 1 mg/ml) in the synovial cavity and daily oral dose (6.2 mg/Kg) of 6-shogaol for 28 days. C (Mag × 10), D (Mag × 20), scale bars = 20 μm. Arrows indicate smooth articular cartilage surface and regular arrangement of chondrocytes in isogenous groups.

After 28 days of exposure of the two groups of rats to a single CFA injection in the synovial cavity of the knee of each rat and daily oral doses of either peanut oil (control group) or 6-shogaol, histological sections of the knee joint were prepared. Figure 4 shows a representative histological section of the knee joint with significant cartilage damage that was produced in the peanut oil treated control group of rats. But no such damage of the cartilage was detected in the 6-shogaol treated group of rats (fig. 4). In fact, the treatment with 6-shogaol was associated with preservation of the morphological integrity of the cartilage lining the femur as shown in the representative micrograph (fig. 4).

## Discussion

Within 2 days of injecting 0.5 ml (1 mg/ml) complete Freund's adjuvant (CFA) into the synovial cavity of the right knees of three groups of rats, localized edematous swelling to a maximum magnitude was produced as the first sign of inflammatory response. The volume of fluid injected in the knee was discounted from this response by using the knee circumference taken thirty minutes after the injection as the baseline from which the magnitude of edematous swelling was calculated. The time course of maximum edema production after CFA injection was similar to that observed in the study reported by Yu et al [11]. However, the maximum swelling produced in our study was smaller. This difference in magnitude of the response was most likely due to a difference in potency of the commercial CFA preparation used in our study. Support for this suggestion is based on our use of a larger amount (500 μg) of the commercial CFA compared to the 250 μg prepared and used by Yu et al [11] for induction of monoarthritis in the knee. Also, in other studies with the CFA model [8,9] amounts above 250 μg usually produce polyarthritis which was not obtained in our study; therefore our commercial preparation must have been of a lower potency.

During the initial phase of the inflammatory response, swelling of the knee was greatest on day 2 in the indomethacin treated group compared to the peanut oil treated control group of rats. But, in the 6-shogaol treated group of rats, the magnitude of swelling was significantly lower compared to either of the preceding groups of rats. This difference in the early response to indomethacin treatment compared to its usual anti-inflammatory effect, appeared to have resulted from increased production of pro-inflammatory cysteinyl leukotrienes whose effect was inhibited in this study by the receptor antagonist, montelukast [22]. This pro-inflammatory effect was also obtained with the use of other non-steroidal anti-inflammatory drugs (NSAIDs). Aspirin, for example, was reported to increase the production of cysteinyl leukotrienes and precipitate an asthmatic attack in susceptible subjects [23,24]. The underlying mechanism of the NSAIDs effect was postulated as resulting from an increase in production of leukotrienes from the common arachidonic acid substrate following inhibition of cyclooxygenase and production of prostaglandins by aspirin and indomethacin [23,25].

After the early inflammatory response on day 2, indomethacin and 6-shogaol progressively reduced the edematous swelling of the knee to a level close to the pretreatment value by day 28. In contrast, maximum swelling of the knee in the peanut oil treated controls was maintained up to the end of the investigational period on day 28. These results suggest that in this model of inflammation, 6-shogaol (6.2 mg/Kg/day) reduced edematous swelling of the knee with similar effectiveness as an equivalent human daily dose of indomethacin (per Kg body weight = 2–3 mg/day) [26], which is a standard anti-inflammatory drug [26]. Note that although enterohepatic cycling was previously reported by Oberbauer et al [27] as contributing to delayed onset of indomethacin therapeutic effect, this action may have only contributed a minor effect compared to the cysteinyl leukotriene involvement in the early exaggeration of the CFA inflammatory response observed in our study.

The effectiveness of 6-shogaol and indomethacin in reducing the CFA-induced edematous swelling of the knee was associated with significant reduction of total leukocyte migration as well as lymphocytes and monocytes/macrophages migration from the blood into the synovial cavity. These inflammatory cells are major contributors to the initiation and maintenance of the immune response [28,29], and their concentrations were significantly greater in the peanut oil treated controls compared to either the 6-shogaol or indomethacin treated group of rats in this study. Regulation of migration of these cells from the blood was reported to be facilitated by adhesive interactions with the vascular endothelium [18]. Soluble vascular cell adhesion molecule, type-1 (soluble VCAM-1) was identified as a major mediator of the adhesive interactions [19,30]. In our study, the 6-shogaol treated group of rats had significantly lower concentration of soluble VCAM-1 in the blood and leukocyte infiltration into the synovial cavity compared to the peanut oil treated control group. The relationship of these two variables showed strong positive correlation in which the magnitude of leukocyte infiltration into the synovial cavity was dependent on the concentration of soluble VCAM-1 produced in the blood.

It is also interesting to note that whereas low infiltration of inflammatory cells in the synovial cavity of the 6-shogaol treated rats was not associated with damage to the cartilage, greater infiltration of inflammatory cells obtained in the peanut oil treated controls was associated with damage to the cartilage in these rats. This type of damage in human arthritic joints was previously reported to result from cellular output of toxic agents such as nitric oxide and its oxidizing product, peroxynitrite which was detected by the presence of protein nitrotyrosine marker in synovial tissues of rheumatoid and osteoarthritic patients [31]. Free radicals were also reported to be involved in producing damage to the arthritic joint. Examples include malondialdehyde (MDA) which was detected in high concentration in the blood of arthritic patients [32], and products of hydrogen peroxide (hydroxyl radical and hypochlorous acid produced by enzymatic action of myeloperoxidase) which were associated with adjuvant arthritis in rats[6].

In a more recent study of extracts from two species of ginger (*Alpinia galanga and Zingiber officinale*) on human synoviocytes, the investigators reported that the extracts decreased TNF_α _induced production of macrophage chemotactic factor (MCP-1) and interferon-γ activated protein (IP-10) as well as their respective mRNA [33]. In another study of crude and fractionated extracts of *Zingiber officinale *on monocyte production of TNF_α _and PGE_2_, the investigators reported that both the crude and fractionated extracts reduced production of these agents. However, although the shogaol-rich extract inhibited the production of PGE_2 _from the cells, they had no effect on the induction of COX-2 enzyme [34]. The implication of these results is that compounds such as 6-shogaol derived from ginger has the potential to inhibit the production of tissue damaging agents such as TNF_α _as well as chemotactic factors that would attract inflammatory cells to areas of joint inflammation.

When these results obtained by Phan et al [33] and Lantz et al [34] are considered together with our findings, there is strong support for anti-inflammatory properties for 6-shogaol and other compounds derived from ginger. 6-Shogaol was particularly effective in our study in reducing CFA induced chronic inflammation in the knee joint of rats. Its effectiveness was reflected as reduction of edematous swelling of the joint, mononuclear cell infiltration that was dependent on soluble VCAM-1 concentration in the blood and prevention of cartilage damage.

## Conclusion

It is concluded from the results of this study that 6-shogaol reduced CFA induced chronic inflammatory response in the knee joint of rats and protected the femoral cartilage from damage. These findings suggest that 6-shogaol has useful anti-inflammatory properties that can be exploited.

## Methods

### Experimental animals

Female Sprague-Dawley rats (weighing 200–250 g) were used in this study. They were bred from Taconic farm stock (USA) in the Animal Care facility of the Department of Basic Medical Sciences, University of the West Indies. Throughout the experimental period, the animals were housed three per cage and kept on a 12:12-hour light-dark cycle. They also had access to food (PMI formulab 5008, labdiet) and water *ad libitum*. Procedures involving animal care and use conformed to the guidelines of the National Research Council (USA) for the use and care of laboratory animals (ILAR/NRC, 1996).

### Induction of chronic inflammation and other treatments

The rats were anesthetized with ether to facilitate the injection of 0.5 ml (1 mg/ml) of commercially prepared complete Freund's adjuvant (CFA from Sigma Aldrich) into the synovial cavity of the right knee of each rat. Note that this was the minimum amount of the CFA preparation that produced measurable inflammatory response in our study. After recovery from the anesthetic, the rats were allowed to develop chronic inflammation over a period of 28 days. During this period and one day before CFA injection, each animal (in groups of 6) received a daily oral dose (via gavage) of either 0.2 ml peanut oil (Hain Celestial, USA) in the control group or 6.2 mg/Kg 6-shogaol (Chromadex, California) in 0.2 ml peanut oil in the test group or 2 mg/Kg indomethacin (Sigma Aldrich) which was used as a standard drug to show the typical anti-inflammatory effect on the edematous swelling of the knee.

### Measurement of edematous swelling of the knee

Throughout the investigation period, a flexible tape was used in accordance with the procedure reported by Yu et al [11] to make daily measurement of the knee circumference at marked points. Note that in order to discount any volume change due to injection of the 0.5 ml CFA, the base line circumference of the knee for determination of inflammatory response was taken thirty minutes after injection of CFA. The measurement was done in triplicate by a technician who was not involved in the study, and the average of these measurements was used as the knee circumference. The daily change in knee circumference was determined from these measurements and compared graphically for an indication of the effects of the treatments on the magnitude of edematous swelling of the knee.

### Determination of leukocyte concentration in synovial fluid

Leukocyte concentration in the synovial fluid was determined on day 28 of the investigational period when 6-shogaol was most effective in reducing the swelling of the knee. The protocol used for this determination involved anesthetization (with 0.8 ml/100 g of 15% urethane, intraperitoneally) of each rat in the peanut oil treated group and the 6-shogaol treated group. This was followed by an incision of the skin overlying the anterior aspect of the right knee and insertion of a 26 gauge hyperdermic needle into the synovial cavity. This arrangement facilitated the infusion of 250 μL 0.9% saline into the synovial cavity over a period of 2 minutes. A second hyperdermic needle (26 gauge), which served as an outflow cannula, was inserted into the synovial cavity approximately 3 mm from the infusion needle. Five minutes after infusion of all the saline, 200 μL of fluid was withdrawn from the synovial cavity over a period of two minutes. This synovial fluid was then used in a Coulter counter (Coulter Corporation) to determine the concentrations of total leukocytes, lymphocytes and monocytes/macrophages in accordance with the procedure of Salinas [20].

### Determination of soluble VCAM-1 concentration in blood samples

On day 28 of the investigational period, when 6-shogaol was most effective in reducing the swelling of the knee, 2 ml of blood was taken from each rat in the peanut oil treated group and the 6-shogaol treated group via vacupuncture of the heart. Each sample of blood was centrifuged (1500 REVS/10 mins) to obtain the separated plasma which was stored at -20°C until it was assayed for soluble VCAM-1. A microplate reader (MF iEmS, Labsystems) was used to carry out this assay by Enzyme Immunoassay analysis (EIA) in accordance with the procedures of the EIA kit supplied by the manufacturer (US Biological).

### Preparation of histological sections of the knee joint

After obtaining blood samples for quantification of soluble VCAM-1 and withdrawal of fluid from the synovial cavity for determination of leukocyte concentration on day 28, each rat in the peanut oil treated group and 6-shogaol treated group was sacrificed with an overdose of ether. The total knee joint was removed surgically and fixed for 4 days in 4 % formaldehyde (Mallinckrodt Backer Inc.) followed by decalcification in formic acid (BDH Chemicals Ltd.) according to the procedure by Joosten et al [21]. Subsequent to these treatments of the knee joint, it was dehydrated with progressively increasing concentrations (70% – absolute) of alcohol (BDH Chemicals Ltd.) and embedded in paraffin. From each paraffin preparation, longitudinal sections (5 μm) were cut and then stained with either hematoxylin (Hopkin and Williams Ltd.) and eosin (BDH Chemicals Ltd) or safranin O (Sigma Chemical Company) in accordance with the procedures described by Joosten et al [21]. Each stained section was examined with a light microscope for damage to the articular cartilage lining the head of the femur.

### Statistical analysis

Each value was expressed as mean ± SEM, and n refers to the number of animals used. The unpaired Student's t test was used for making statistical comparisons of the results between the control group and the test group, and a value of p ≤ 0.05 was used to define statistically significant differences. The Pearson correlation coefficient was used to determine the relationship between the concentration of soluble VCAM-1 and the magnitude of leukocyte infiltration in the synovial cavity.

## Abbreviations

BDH – British Drug House

CFA – Complete Freund's Adjuvant

CGRP – Calcitonin gene-related peptide

COX-2 – Cyclooxygenase -2

EIA – Enzyme Immunoassay

ILAR/NRC – Institute for Laboratory Animal Research/National Research Council

IP-10 – Interferon inducible protein 10

mm – Millimeter

MDA – Malondialdehyde

MCP-1 – Macrophage chemotactic factor -1

mRNA – Messenger ribonucleic acid

NSAIDs – Non-steroidal anti-inflammatory drugs

p – Statistical Probability

PGE_2 _– Prostaglandin E_2_

PMI – Purina Mills Incorporated

REVS – Revolutions

SP – Substance P

SEM – Standard error of the mean

TNF_α _– Tumor necrosis factor-α

VCAM-1 – Vascular cell adhesion molecule-1

## Authors' contributions

AL conducted experiments with 6-shogaol and prepared figures and the manuscript. JS conducted experiments with indomethacin. MG assisted with the preparation and analysis of histological sections. OS prepared the manuscript. All authors read and approved the final manuscript.

## References

[B1] Zarate RS, Yeoman MM (1992). Application of two rapid techniques of column chromatography to separate the pungent principles of ginger (*Zingiber officinale Roscoe*). J of Chromatography.

[B2] Suekawa M, Isige A, Yuasa K (1984). Pharmacological studies on ginger I. Pharmacological actions of pungent constituents, 6-gingerol and 6-shogaol. J Pharmacobiodyn.

[B3] Suekawa M, Yuasa K, Isono M, Sone H (1986). Pharmacological studies on ginger IV. Effect of 6-shogaol on the arachidonic cascade. Nippon Yakurigaku Zasshi.

[B4] Srivastava KC, Mustafa T (1992). Ginger (Zingiber officinale) in rheumatism and musculosketal disorders. Med Hypotheses.

[B5] Bliddal H, Rosetzsky A (2000). A randomized placebo controlled cross-over study of ginger extracts and ibuprofen in osteoarthritis. Osteoarthritis Cartilage.

[B6] Santos L, Tipping PG (1994). Attenuation of adjuvant arthritis in rats by treatment with oxygen radical scavengers. Immunol Cell Biol.

[B7] Bendele AM (2001). Animal models of rheumatoid arthritis. J Musculoskel Neuron Interact.

[B8] Shenker N, Haigh R, Roberts E, Mapp P, Harris N, Blake D (2003). A review of contralateral responses to a unilateral inflammatory lesion. Rheumatology.

[B9] Donaldson LF, Seckl JR, McQueen DS (1993). A discrete adjuvant-induced monoarthritis in the rat: effects of adjuvant dose. J Neurosci Methods.

[B10] Butler SH, Godefroy F, Besson JM, Weil-Fugazza J (1992). A limited arthritic model for chronic pain studies in the rat. Pain.

[B11] Yu YC, Koo ST, Kim CH, Lyu Y, Grady JJ, Chung JM (2002). Two variables that can be used as pain indices in experimental animal models of arthritis. Journal of Neuroscience Methods.

[B12] Allison AC (1979). Mode of action of immunological adjuvants. J Reticuloendothelial Soc.

[B13] Stills HF, Bailey MQ (1991). The use of Freund's Complete Adjuvant. Lab Animal.

[B14] Stewart-Tull D (1995). Freund-type mineral oil adjuvant emulsions. the Theory and Practical applications of adjuvants.

[B15] Volin MV, Szekanecz Z, Halloran MM (2000). PECAM-1 and leukosialin (CD43) expression correlate with heightened inflammation in rat adjuvant-induced arthritis. Experimental and Molecular pathology.

[B16] Sfikakis PP, Tsokos GC (1997). Clinical use of the measurement of soluble cell adhesion molecules in patients with autoimmune rheumatic disease. Clinical and Diagnostic Labaratory Immunology.

[B17] Frenette PS, Wagner DD (1996). Adhesion molecules Part 1. The New England J of Medicine.

[B18] Carter RA, Campbell K (2002). Vascular cell adhesion molecule-1(VCAM-1) blockade in collagen-induced arthritis reduces joint involvement and alters B cell trafficking. Clin Exp Immunol.

[B19] Kitani A, Nakashima N (1998). Soluble VCAM-1 induces chemotaxis of jurkat and synovial fluid T cells bearing high affinity very late antigen-4. The Journal of Immunology.

[B20] Salinas M (1997). Comparison of manual and automated cell counts in EDTA preserved synovial fluid. Storage has little influence on the results. Ann Rheum Dis.

[B21] Joosten LAB, Lubberts E, Helsen MA (1999). Protection against cartilage and bone destruction by systemic 1L-4 treatment in established murine type II collagen induced arthritis. Arthritis Research.

[B22] Yu G-L, Wei E-Q, Zhang S-H, Xu H-M, Chu L-S, Zhang W-P, Zhang Q, Chen Z, Mei R-H, Zhao M-H (2005). Montelukast, a cysteinyl leukotriene receptor-1 antagonist, dose-and time-dependently protects against focal cerebral ischaemia in mice. Pharmacology (International Journal of Experimental and Clinical Pharmacology).

[B23] Cowburn AS, Sladek K, Soja J, Amadek L, Nizankowska E, Szczeklik A, Lam BK, Penrose JF, Austen KF, Holgate ST, Sampson AP (1998). Overexpression of leukotriene C_4 _synthase in bronchial biopsies from patients with aspirin-intolerant asthma. J Clin Invest.

[B24] Sousa AR, Parikh A, Scadding G, Corrigan CJ, Lee TH (2002). Leukotriene-receptor expression on nasal mucosal inflammatory cells in aspirin-sensitive rhinosinusitis. N Engl J Med.

[B25] Empey LR, Walker K, Fedorak RN (1992). Indomethacin worsens and a leukotriene biosynthesis inhibitor accelerates mucosal healing in rat colitis. Can J Physiol Pharmacol.

[B26] Wagner W, Khanna P, Furst DE (2004). Non steroidal anti-inflammatory drugs, disease modifying anti-rheumatic drugs, non-opioid analgesics and drugs used in gout. Basic and Clinical Pharmacology.

[B27] Oberbauer R, Krivanek P, Turnheim K (1993). Pharmacokinetics of indomethacin in the elderly. Clin Pharmacokinetics.

[B28] Mapp PI, Grootveld MC, Blake DR (1995). Hypoxia, oxidative stress and RA. BR Med Bull.

[B29] Weyand CM (2000). The role of T cells in RA. Arch Immun Ther Exp.

[B30] Tokuhira M, Hosaka S, Volin MV (2000). Soluble VCAM-1 mediation of monocyte chemotaxis in RA. Arthritis Rheum.

[B31] Sandhu JK, Robertson S, Birnboim HC, Goldstein R (2003). Distribution of protein nitrotyrosine in synovial tissues of patients with rheumatoid arthritis and osteoarthritis. J Rheumatol.

[B32] Vasanthi P, Ganesan N, Hariprasad C, Rajasekhar G, Meera S (2004). Plasma lipophilic antioxidant and pro-oxidant levels in rheumatoid arthritis. J Indian Rheumatol Assoc.

[B33] Phan PV, Sohrabi A, Polotsky A, Hungerford DS, Lindmark L, Frondoza CG (2005). Ginger extract components suppress induction of chemokine expression in human synoviocytes. Journal of Alternative and Complementary Medicine.

[B34] Lantz RC, Chen GJ, Sarihan M, Solyom AM, Jolad SD, Timmermann BN (2006). The effects of extracts from ginger rhizome on inflammatory mediator production. Phytomedicine.

